# Hydrogel increases localized transport regions and skin permeability during low frequency ultrasound treatment

**DOI:** 10.1038/srep44236

**Published:** 2017-03-13

**Authors:** Tatiana Aparecida Pereira, Danielle Nishida Ramos, Renata F. V. Lopez

**Affiliations:** 1 University of Sao Paulo, School of Pharmaceutical Sciences of Ribeirao Preto, Ribeirao Preto, SP, Brazil

## Abstract

Low frequency ultrasound (LFU) enhances skin permeability via the formation of heterogeneous localized transport regions (LTRs). In this work, hydrogels with different zeta potentials were used as the coupling medium for LFU to investigate their contribution to LTR patterns and to the skin penetration of two model drugs, calcein and doxorubicin (DOX). When hydrogels were used, LTRs covering at least a 3-fold greater skin area were observed compared to those resulting from traditional LFU treatment and sodium lauryl sulfate. More LTRs resulted in an enhancement of calcein skin permeation. The zeta potential of the hydrogels affected the skin penetration of the positively charged DOX; the cationic coupling medium decreased the DOX recovered from the viable epidermis by 2.8-fold, whereas the anionic coupling medium increased the DOX accumulation in the stratum corneum by 4.4-fold. Therefore, LFU/hydrogel treatment increases LTRs areas and can target ionized drugs to specific skin layers depending on the zeta potential of the coupling medium.

In recent decades, low frequency ultrasound (LFU) has been studied extensively in transdermal drug delivery^1^,^2^,^3^,^4^,^5^,^6^,^7^,^8^. It is known that the pre-treatment of the skin with LFU increases skin permeability to several drugs, including therapeutic macromolecules^9^,^10^,^11^. However, skin permeability enhancement is not homogeneous^12^,^13^,^14^,^15^, and it particularly occurs in localized regions of the skin known as localized transport regions (LTRs)^3^,^12^,^13^,^14^,^16^,^17^. At 20 kHz, the most studied frequency in LFU transdermal drug delivery, only 5–10% of the treated skin surface area results in LTR formation, even when long treatment times are used^6^,^12^,^14^,^18^. LTRs are 80 times more permeable than non-LTRs^15^; therefore, a great challenge in LFU treatment is to increase LTR formation with a more homogeneous distribution throughout the skin to increase the permeabilization efficiency.

It is believed that the medium where LFU is applied, i.e., the coupling medium, plays an important role in LTR distribution, as the microjet collapse of cavitation bubbles at the skin surface is the most likely mechanism of LTR formation^3^,^4^,^5^,^6^. Therefore, modifications in the coupling medium to change bubble nucleation has been attempted to increase LTR formation and skin permeability^7^,^13^,^19^,^20^,^21^,^22^.

The most common modification of the coupling medium is the addition of 1% of the surfactant sodium lauryl sulfate (SLS) in a buffer solution. LFU treatment using this coupling medium is known as LFU/SLS^6^,^13^,^16^,^19^. Although LFU/SLS did not significantly increase the total area of the skin covered by LTRs, SLS stabilizes cavitation bubbles, resulting in a population of smaller bubbles^20^,^23^. The smaller cavitation bubbles are likely responsible for a better distribution of LTRs throughout the skin surface. In addition, LFU/SLS results in the formation of LTRs at regions other than below the ultrasound transducer, as occurs when only LFU is applied^16^. Addition of a porous resin in the coupling medium to act as a cavitation nucleus^21^ or of solid particles larger than the threshold of the bubble resonant size (200 μm for an acoustic field of 20 kHz)^22^ were also strategies that have been evaluated with the purpose of increasing the number of LTRs and skin permeability. A recent approach^7^ showed that bubble nucleation and cavitational activity could be greatly increased by the simultaneous application of high frequency ultrasound (HFU) and LFU. The authors found that this association increased both LTR formation and permeation of 4 kDa dextran *in vitro*. The *in vivo* LTR formation was in good agreement with the *in vitro* results^8^.

In this work, we hypothesized that the percentage of LTRs covering the skin surface could be increased by simply using hydrogels as a coupling medium. Although hydrogels are often used in HFU as a coupling medium^24^,^25^, there are no reports in the literature of their use in LFU.

The use of semisolid hydrogels as a coupling medium in LFU could be interesting to study due to (i) the ease of application to the skin; (ii) the energetics of the cavitation bubbles in a viscous medium, which should be high due to higher interfacial tension compared to a liquid coupling medium; and (iii) the dissolution rate of the cavitation bubbles in the viscous medium, which may be slower than in a low viscosity medium; this difference would cause cavitation bubbles formed during an ultrasound duty cycle to be present in subsequent cycles, thereby increasing the number of bubbles and the action of the LFU in skin disruption.

In addition to the effect of the coupling medium on ultrasound-related phenomena^23^, components of the coupling medium themselves may penetrate the skin when LFU is applied. This action could alter the chemical composition of the stratum corneum (SC), thereby contributing to physical modifications caused by LFU in skin permeability. The use of LFU/SLS, for instance, significantly increases the non-LTRs permeability compared to LFU treatment alone because of the known disorganization caused by SLS in the SC^6^,^23^,^26^. Based in these findings, we believe that the presence of the coupling medium components within the SC may modify the penetration of drugs that strongly interact with the SC, thereby increasing the skin permeability to drugs.

Modification of the SC composition by components of the formulation that penetrate the skin under the influence of a physical method has also been investigated. Iontophoresis of a cationic chitosan solution decreased the skin penetration of the positively charged doxorubicin hydrochloride (DOX), which is a drug known for its strong interaction with the SC^27^, compared to treatment with a non-ionic polymeric solution^28^. Thus, it would be interesting to study how the zeta potential of coupling medium hydrogels associated with LFU could modify the interaction of DOX with the SC and thus change the drug skin penetration.

Therefore, the objectives of this study were 1) to determine the contribution of cationic, anionic and non-ionic hydrogels in the formation and distribution of LTRs on the skin surface when used as coupling media in LFU application and 2) to verify the impact of these coupling media on LFU in DOX skin penetration.

## Results

### Physicochemical characterization of the coupling media

Four hydrogels were evaluated as coupling media. Two of them were prepared with non-ionic polymers, poloxamer and hydroxyethyl cellulose (HEC). A third was prepared with the cationic polymer chitosan, and the fourth was prepared with a negatively charged polymer, carbopol. Table 1 shows the zeta potential of the hydrogels and of the SLS solution conventionally used as the LFU coupling medium.

According to Table 1, dispersions composed of poloxamer or HEC can be classified as non-ionic, those composed of chitosan are cationic, and those composed of an SLS solution or carbopol are anionic. The zeta potentials of the hydrogels selected for this study were deliberately different to investigate their influence on drug transport through the skin after LFU treatment. Additionally, the viscosity of all of the hydrogels was on the same order of magnitude in an attempt to control for the influence of the viscosity among the gels in cavitation-bubble formation. The mean viscosity values of the SLS solution and hydrogels are as presented in Table 2.

Viscosity is an important parameter to be determined because it can suppress cavitation^29^. Therefore, it is important to ensure that the energy threshold to the occurrence of cavitation be reached when semisolid hydrogels are used as coupling media. The minimum energy necessary for cavitation to occur into the castor oil coupling medium (0.63–0.89 Pa.s) was 1.6 W/cm^2^ 29. Although hydrogels used in the present work are approximately 3-fold more viscous than the castor oil, they are composed of polymeric networks formed in a highly aqueous environment; more than 80% of the composition of the hydrogels is water. In addition, the energy used in the LFU treatment in the present study was 9.06 ± 0.5 W/cm^2^, 5.5-fold higher than the threshold intensity for cavitation occurrence previously reported for castor oil. Moreover, cavitation is a macroscopically clearly visible effect, and it was observed in the hydrogels coupling medium when LFU was applied.

In addition to viscosity, interfacial tension can also play a significant role in determining the extent of skin permeability enhancement observed as a result of the ultrasound treatment. It can influence, for instance, the size and collapse rate of the cavitation bubbles when ultrasound is applied^30^.

To investigate the influence of the interfacial tension of the hydrogels used as the coupling medium in LTRs formation, two nonionic hydrogels, poloxamer and HEC, with different interfacial tension (Table 2), were selected. Poloxamer is a synthetic surfactant composed by amphiphilic block copolymers, namely poly(ethylene oxide)–poly (propylene oxide)–poly (ethylene oxide) (PEO–PPO–PEO). Like the SLS, it is known that poloxamer can reduce the surface tension of the media. Indeed, the poloxamer hydrogel showed statistically significantly lower interfacial tension among the hydrogels used as coupling medium (Table 2).

### The influence of the coupling medium on LTRs formation

Skin samples were treated with LFU operating at 20 kHz according to previously published methods^6^,^13^,^15^,^18^,^26^. The hydrogels and the SLS solution were used as the coupling media. After treatment, the skin was exposed to an allura red solution to stain LTRs that were subsequently imaged using a digital camera^6^. Table 2 shows the viscosity and interfacial tension of the hydrogels and SLS solution after 1 min of LFU treatment.

Interfacial tension of the SLS solution, HEC, chitosan and carbopol decreased significantly (p < 0.05, t-test), but the decrease was less than 1.5-fold after the LFU treatment. Viscosity, on the other hand, increased 5-fold for poloxamer and decreased 2-fold, 6-fold and 3-fold for SLS solution, HEC and chitosan hydrogel respectively. The most drastic decrease was observed for carbopol hydrogel which viscosity decreased by 10-fold.

**Figure 1:**
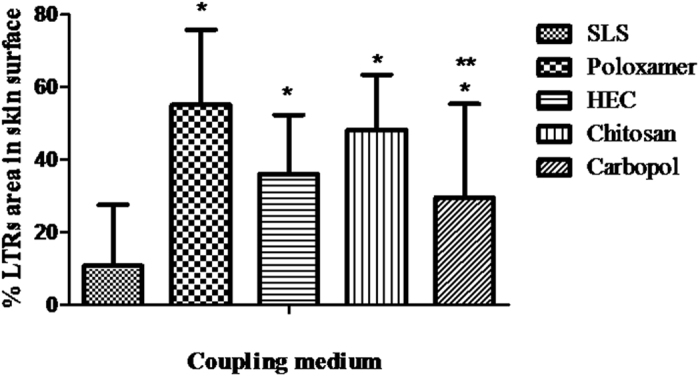
Percentage of LTRs covering the skin surface as a result of the LFU treatment using an SLS solution and different hydrogels as the coupling medium. The error bars represent the standard deviation (n = 10 determinations for 3 different hydrogel samples). *p < 0.05 vs. SLS solution, **p < 0.05 vs. Poloxamer hydrogel. The results were analyzed according to ANOVA, followed by post hoc analysis using Tukey’s test with p < 0.05 as the minimum level of significance.

Figure 1 shows the percentage of LTRs covering the skin surface as a result of the LFU treatment using the SLS solution and the different hydrogels as the coupling medium.

Skin samples treated with LFU using the SLS solution as the coupling medium showed 11 ± 17% of the skin covered by LTRs, whereas treatment using hydrogels significantly increased the LTR formation on the skin surface (p < 0.05) (Fig. 1). Among the coupling medium hydrogels, LFU treatment with poloxamer (LFU/Poloxamer) showed the greatest coverage area of 55 ± 21%, whereas the treatment with carbopol (LFU/Carbopol) resulted in the smallest LTR area (30 ± 26%).

**Figure 2:**
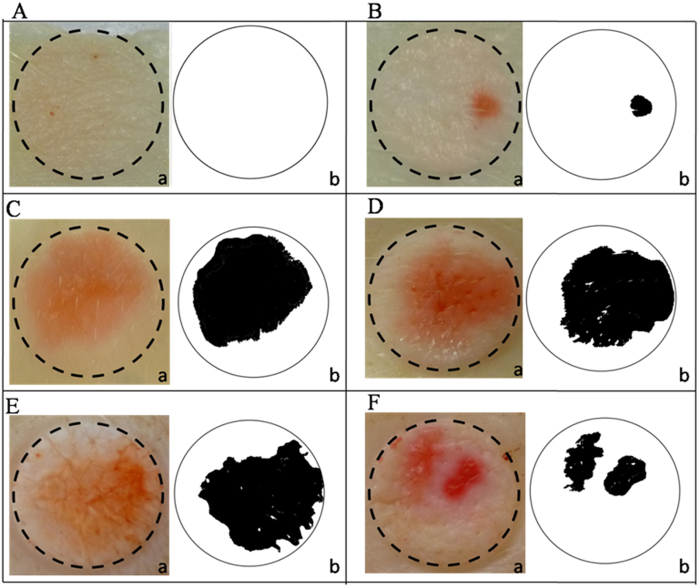
Representative images of the skin treated with LFU and different coupling medium (**A**) untreated skin, (**B**) SLS solution, (**C**) Poloxamer, (**D**) HEC, (**E**) Chitosan and (**F**) Carbopol. (a) Native images of the area of the skin exposed to the dye Allura red. (b) Binary image of the interest region marked by the dotted circle and quantified using imageJ software.

Figure 2 shows representative images of the LTRs formed in skin samples treated with LFU/SLS and with different hydrogels (LFU/hydrogels).

It is possible to observe in Fig. 2B that a single LTR was formed on the skin surface treated with LFU/SLS. However, when the samples were treated with LFU/Poloxamer, LFU/HEC or LFU/Chitosan (Fig. 2C,D and E, respectively), contiguous LTRs were formed on the skin surface. Skin samples treated with LFU/Carbopol (Fig. 2F) showed a more heterogeneous pattern, with LTRs spread in some localized parties of the skin.

### **C**alcein permeability of the skin

**Figure 3:**
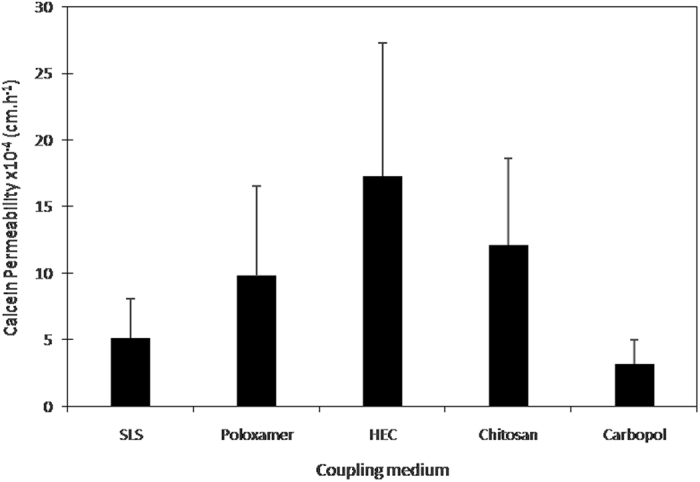
Calcein skin permeability (P) at the steady state. The studies were conducted using the same LFU parameters (9.6 ± 0.5 W/cm^2^, duty cycle, 50% (5 s on, 5 s off); tip displacement, 3 mm. The results were expressed as the mean ± standard deviation (n = 4 determinations for the same initial formulation).

To evaluate the influence of the percentage of LTRs in drugs permeation through the skin, after treatment with hydrogels and the SLS solution, a 0.2% calcein in PBS was placed in contact with the skin. Figure 3 shows calcein skin permeability (P) at the steady state.

Calcein skin permeability was similar after the LFU treatment with non-ionic (poloxamer and HEC) and chitosan hydrogels (ANOVA, followed by Tukey’s post hoc test with p < 0.05 level of significance), but tends to be twice as low after the treatment with the anionic carbopol and SLS solution (Fig. 3). For the hydrogel treatments, this result is consistent with the smaller LTR percentage formed after LFU/Carbopol which was almost 2-fold smaller than other hydrogel treatments (Fig. 1). However, the calcein permeability for the LFU/SLS treatment was not proportional to LTR formation, which occupied a 5-fold smaller area than non-ionic and cationic hydrogel treatments.

### DOX skin permeation

**Figure 4:**
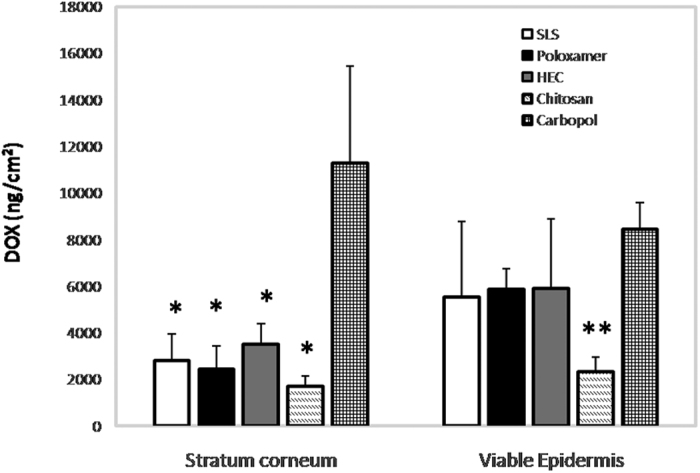
DOX delivered to the skin after LFU treatment using hydrogels as coupling medium. The results were expressed as the mean ± standard deviation (n = 6 determinations for the same initial formulation). The results were analyzed according to ANOVA followed by post hoc analysis using Tukey’s test with p < 0.05 as the minimum level of significance. SC: *p < 0.05 vs. Carbopol; Viable epidermis: **p < 0.05 vs. Carbopol.

The influence of the zeta potential of the coupling medium on the skin permeation after LFU treatment was evaluated using a drug that strongly interacts with the SC, DOX. DOX recovered from the SC and viable epidermis after 26 h of permeation experiments through the skin treated with LFU/hydrogels and SLS solution can be observed in Fig. 4.

In the SC, treatment of the skin with LFU/Carbopol resulted in at least 3-fold more DOX penetration than treatment with the other hydrogels and SLS solution (p < 0.05). Although not statistically significant, lower DOX penetration into the SC was obtained when chitosan was used as a coupling medium. No changes were observed in DOX penetration when the non-ionic coupling medium, poloxamer and HEC hydrogels, were used (Fig. 4). Treatment with the anionic SLS solution increased DOX penetration, but the penetration was less than the LFU/Carbopol treatment. The DOX penetration was similar to that achieved with the LFU/non-ionic hydrogel treatment. Percentage of LTRs, however were 3-fold smaller for LFU/SLS when compared to the LFU/Carbopol treatment (Fig. 1).

The SC, composed of dead cells is the main skin barrier. Although drug penetration into the SC is interesting as it serves as a drug depot, most skin diseases, such as skin cancer affect the viable skin layers. Therefore, it is important that topical treatments facilitates the drug delivery to the viable epidermis. In the viable epidermis, the tendency of LFU/Chitosan to lower DOX penetration is clear. The LFU/Chitosan treatment decreased DOX penetration in the viable skin by approximately 2.5-fold when compared with the LFU treatment with the non-ionic coupling media and the SLS solution and by 3.6-fold when compared to the treatment with LFU/Carbopol.

The ratio viable epidermis/SC of DOX penetration was about 2 for non-ionic hydrogels and the SLS solution treatment, 1.4 for Chitosan and 0.7 for Carbopol, suggesting that LFU/Carbopol treatment facilitates DOX interaction within the SC but its diffusion into the viable epidermis when compared with the others treatment was impaired.

To determine the amount of DOX that permeate through the skin, the receptor solution was analyzed at the end of all experiments, however DOX was not detected in any of the receiver medium (the quantification limit of the analytical method was 100 ng/mL).

## Discussion

Several studies have conclusively shown that LFU skin permeabilization is mediated by transient cavitation, which creates LTRs in the skin surface when skin is pre-treated using 1% SLS as coupling medium (LFU/SLS)^2^,^6^,^7^,^8^. However, the formation of LTRs in the skin surface occurs heterogeneously, covering only approximately 5–10% of the total area of the skin exposed to LFU/SLS^12^,^13^,^15^,^31^. Recently, LFU studies are being used for the development of new strategies to increase LTRs formation and their distribution on the skin surface. The combination of LFU and HFU produced LTRs that covered approximately 30% of the total area of the skin^7^. However, the device used to combine both ultrasound frequencies is not simple for clinical application. The use of hydrogels as coupling medium, as proposed in our work, can be a simple and efficient strategy to improve the percentage and homogeneity of LTRs distributed in the skin surface. More LTRs can result in higher amount of drug penetration in deeper layers of the skin and in more effective treatment of cutaneous diseases. However, permeability of LTRs differs depending on treatment method. Therefore, the skin permeability of two drugs, calcein and DOX, were evaluated in this work to study the influence of hydrogels in LTRs permeability.

Interfacial tension of the coupling medium can play significant role in the extent of skin permeability enhancement as a result of the ultrasound treatment. A medium with a low surface tension can lead to the formation of large population of small cavitation bubbles, which, although less energetic, are more stable against coalescence^23^,^32^. Therefore, theoretically, the use of coupling media with low surface tension can improve the extent of skin area covered by LTRs due to the increased number of cavitation bubbles; however, the collapse of these bubbles may be less energetic microjets than those produced in a high interfacial tension medium. Furthermore, the increase in the coupling medium temperature caused by LFU application^6^ decreases the interfacial tension^33^ which could have facilitated more the formation of small cavitation bubbles. Indeed, this decrease was observed in the formulations after 1 min of LFU treatment (Table 2). However, in the experiments performed, the decrease in interfacial tension did not correlate to the number of LTRs. Carbopol hydrogel, for instance, which has an interfacial tension similar to HEC and chitosan (Table 2), showed a lower percentage of LTRs (Fig. 1). The treatment with SLS, whose solution present the lowest interfacial tension, resulted in the lowest percentage of LTR.

The increase in the LTRs percentage may thus be related with the viscosity of the dispersions when LFU was applied (Table 2). Poloxamer, an *in situ* forming gel that has a sol-gel transition temperature of 23.0 ± 0.4 °C^34^, showed an increase in viscosity likely related to the temperature rise when LFU was applied (Table 2). LFU/Poloxamer treatment resulted in the highest percentage of LTRs (Fig. 1). Among the hydrogels, carbopol, having the lowest viscosity after LFU application, showed the lowest LTR percentage. Further studies with hydrogels of the same polymer but with different degrees of polymerization and crosslinking agents should be evaluate to understand better the influence of viscosity in LTRs formation.

The use of hydrogels as a coupling medium in LFU treatment resulted in a high number of LTRs covering the skin surface when compared with the use of other coupling medium described in the literature. Compared to the LFU/SLS treatment, the skin area occupied by LTRs was approximately 3 to 5-fold larger (Fig. 1). Compared to the strategy of associating LFU and HFU^7^,^8^, LFU/Poloxamer showed an LTR area of approximately 1.6-fold higher.

However, it is not only LTRs that influence the permeability of DOX into the skin. As pointed out earlier, permeability could also be due to the components of the coupling medium which can enter the skin and alter the characteristics of both the LTRs and other regions of the skin. The polymers which make up the hydrogels used in the coupling medium can therefore alter the permeability of the skin differently.

To verify whether the increase of LTR area resulted in an increase in drug penetration through the skin, calcein was used as a model drug. Calcein is a hydrophilic fluorophore that is negatively charged and is unable to passively permeate the skin to a significant extent. Skin treatment with LFU/Poloxamer, covering 55% of the surface of the skin with LTRs (Fig. 1), resulted in a calcein skin permeability of approximately 8* × *10^−4^ cm/h. On the other hand, skin treatment with LFU/Carbopol, covering approximately 30% of the skin surface with LTRs, resulted in a 2-fold smaller calcein skin permeability. Therefore, higher LTRs number increased the calcein skin permeation, which is in accordance with other experiments performed with LFU^6^,^15^. The LFU/SLS treatment, however, which resulted in the smallest LTRs percentage among the treatments, showed a higher permeability enhancement than LFU/Carbopol. This result is possibly related to the SLS performance even in non-LTRs.

SLS is a known skin penetration enhancer, which has irritant properties and disorganizes the SC^6^,^13^,^35^. Chitosan is also reported as a skin penetration enhancer^36^. Therefore, the higher calcein penetration resulted after LFU/Chitosan treatment (Fig. 3) could be also related to chitosan’s action in the non-LTRs. Based on these results, it is possible to suggest that the addition of a skin penetration enhancer, such as SLS, in the hydrogels could further increase the skin permeability after LFU/Hydrogel treatment. Care should be taken to the addition of a surfactant in the gel as to not destroy the polymer three-dimensional structure and the consequent loss of hydrogel viscosity.

Other hydrogels could also be used as coupling media to take an advantage of their modifications in LTR permeability and potential benefits for the skin. The use of a hydrogel coupling medium composed of hyaluronic acid, a glycosaminoglycan which has been explored for its antiaging properties^37^, could, for instance, result in an improved antiaging activity besides its influence in LTRs formation and permeability. In summary, specific characteristics of the coupling medium polymer could change the affinity of a drug with the skin, modifying the drug penetration or its interactions with different skin layers.

For drugs that are known to interact with the SC by electrostatic mechanisms, the zeta potential of the gels used as coupling media should be taken into consideration. The influence of the zeta potential of the coupling medium in the skin permeation was noticed in the experiments performed with DOX (Fig. 4). DOX is a positively charged drug (pKa = 8.1) with a molecular weight (580 g/mol) similar to the calcein (620 g/mol). However, it is known that DOX strongly interacts with anionic lipids in the SC^28^,^38^,^39^. Figure 4 showed that pre-treatment with LFU and the cationic chitosan coupling medium decreased the amount of DOX recovered from the viable epidermis 2.9-fold. It is likely that chitosan, which is a polysaccharide with polycationic character^40^, penetrated the skin in the LTRs when LFU was applied. The subsequent skin penetration of the positively charged DOX probably encountered LTRs with a cationic character, which may have resulted in an electrostatic repulsion between the DOX and the LTR, thereby decreasing DOX penetration. In addition, the use of another physical method, iontophoresis, to increase DOX skin penetration also showed to suffer influence of chitosan. Iontophoresis uses a low intensity electric current to increase drugs skin penetration^41^,^42^. Its use to delivery DOX from a chitosan solution resulted in a smaller DOX skin penetration when compared to a non-ionic HEC gel^28^.

It is interesting to note that the pre-treatment of the skin using LFU/Carbopol (an anionic coupling medium), which resulted in the lowest percentage of LTRs among the gels (Fig. 1), showed DOX penetration in the viable epidermis similar to that found when non-ionic coupling media with a high percentage of LTRs were used. Furthermore, LFU/Carbopol treatment significantly increased the DOX amount in the SC compared with other hydrogels. It is possible that the negative charge of this polymer left LTRs with a negative potential, which increased DOX interactions via electrostatic attraction to the SC resulting in a deposit of DOX into the SC. However, SLS which is also anionic, increased the amount of DOX in the SC but the increase was less than that of carbopol probably because of its nonpolymeric nature. This way, SLS present less interaction sites with DOX and can also diffuse through the LTRs easily than the carbopol. The percentage of LTRs observed after the LFU treatment with SLS which was smaller than that observed with carbopol should also be considered.

Although LFU/Carbopol treatment was the one that resulted in more DOX penetration into the viable epidermis, the target site of most topical treatments, DOX diffusion rate from SC to the viable epidermis was the smallest when compared with the other treatments. Due to the high molecular weight of the polymer, it is possible that more of this polymer is present in the peripheral part of the LTRs, the SC area, and less in the viable epidermis area. With more carbopol interacting with the SC, DOX diffusion into the deep skin layers may have been impaired, decreasing the epidermis/SC diffusion rate.

Therefore, DOX skin penetration after LFU pre-treatment is related not only to the number of LTRs but also to its interactions with the skin and with the coupling medium. Simple changes in the ionization of the LTRs components, which are dependent on the zeta potential of the coupling medium, can modify the transport of ionized drugs through the skin.

Therefore, the results presented above suggest that the use of LFU with hydrogels as the coupling medium is a simple and effective alternative to enhance the LTR area of the skin. Furthermore, it is possible to target drugs to specific skin layers by tuning the zeta potential of the coupling medium.

## Methods

### Coupling medium preparation

Four hydrogels were evaluated. Two of them were prepared with non-ionic polymers, poloxamer (EmbraFarma, Brazil) and HEC (Galena, Brazil), one was prepared with a cationic polymer, chitosan (medium molecular weight, 190–310 kDa) (Sigma-Aldrich, USA), and one was prepared with a negatively charged polymer, carbopol 940 (Mapric, Brazil).

The cationic hydrogel was prepared by dispersing chitosan (1.5%) in 0.5% acetic acid solution. The negative hydrogel was prepared by dispersing carbopol (0.25%) in water followed by the addition of sodium hydroxide at 1 M to attain pH 7.0. The non-ionic hydrogel composed of HEC was obtained by dispersing HEC (1.0%) in hot water (40 ± 5 °C). The non-ionic hydrogel composed by poloxamer was prepared as previously described^34^. Briefly, poloxamer (20%) was dispersed in cold water (5 ± 2 °C) and kept in the refrigerator for at least 24 h to ensure the complete polymer dispersion.

### Physical chemical characterization of the hydrogels

It was determined by eletrophoretic mobility using a Zetasizer Nano ZS90 (Malvern Instruments, UK). The samples were diluted (1:10) in ultra-purified water, and data analyses were performed using the Helmholtz-Smoluchowski approximation. Rheological measurements were performed using a Rheometer R/S Plus PTR-I with P50-1 spindle using cone and plate geometry (Brookfield, Germany), equipped with Rheo 2000 V.2.8 software. All tests were performed in triplicate. The values of mean viscosity were obtained in Pa.s. All measurements were carried out at 25 ± 2 °C and after 1 min of LFU treatment. Surface tension was measured using a tensiometer (Fisher Scientific model 20, USA) based on the Lecomte du Noüy method using a rigid platinum ring. All measurements were carried out before, at room temperature and after the LFU treatment using the surface tension of purified water (73.3 mN.m^−1^) as a control.

### Preparation and treatment of the skin samples

The animal experiments were performed in accordance with the National Institute of Health’s Guide for the Care and Use of Laboratory Animals and were approved by the ethical committee on animal use of the University of São Paulo– Protocol n°11.1.727.53.1). Skin was harvested from pig ears immediately after slaughter of the animals (OlhosD’ÁguaLtda, Brazil) and was transported to the laboratory at 4 °C. The skin was sectioned in strips that were stored at −80 °C for up to 3 months. Before the experiment, the skin strips were thawed, and the excess of hair was trimmed using surgical scissors. The strips were then dermatomed at 700 μm using a Dermatometer Micromotor 31 S (Nouvag AG, Switzerland) and cut into 20 × 20 mm to be mounted in vertical diffusion cells with a 1.2 cm inner diameter. Before mounting the skin samples in diffusion cells, a 150-μm opening nylon mesh (Sefar Filtration, USA) was placed onto the top of the receiver chamber for mechanical support. The receiver chamber was filled with phosphate buffer saline (PBS; 0.01 M of phosphate, 0.137 M of NaCl), the skin was put over the nylon mesh with the SC facing up, and the donor chamber was filled with the appropriate hydrogel for LFU treatment or penetration studies. Prior to each experiment, the electrical resistivity (R) of the skin was determined according to ref. 6. Any skin with an initial R of 50 K.cm^2^ was considered damaged and was not used in the experiments.

### Treatment of the skin using LFU

LFU treatment of skin samples was carried out according to previously published methods^6^,^13^,^15^,^18^,^26^,^35^. The ultrasound system used was a VCX 500 (Sonics & Materials, USA) operating at 20 kHz. The intensity of the horn was calibrated using calorimetry^19^, and skin pre-treatments were performed using the following identical experimental conditions: intensity, 9.6 ± 0.5 W/cm^2^; duty cycle, 50% (5 s on, 5 s off); tip displacement, 3 mm; and coupling medium composed of (I) 1% SLS in PBS, (II) Poloxamer, (III) HEC, (IV) Chitosan and (V) Carbopol hydrogels. Allura red (Sigma-Aldrich) at 0.025% was added to all coupling media. Samples were treated until reaching an R of 0.7 ± 0.2 KΩ.cm^2^ 18,43. Treatments performed with poloxamer and chitosan hydrogels took 2.3 ± 0.8 min to attain this R, HEC and SLS took 4.6 ± 1 min and carbopol 7.0 ± 1.1 min. After each minute of treatment, the coupling medium was changed to minimize thermal effects, and the skin R was measured to determine whether the target *R* was reached. After treatment, the skin samples were rinsed with PBS to remove the excess coupling medium. Then, the donor chamber was filled with 0.025% of allura red in PBS for 1 h for subsequent imaging of the LTRs^6^.

### Quantification of LTR areas in LFU-treated skin

After 1 h in contact with the allura red solution, the stained skin samples were removed from the diffusion cells, rinsed with PBS and blotted dry. The skin surface was then imaged using a digital camera (Panasonic Lumix, 16 mega pixels, Brazil) positioned at 20 cm above the skin^6^. The images were processed using Adobe Photoshop CS3 according to ref. 7. First, the blue-channel of the image was isolated and the image was cropped to capture only the image of the portion of the skin exposed to allura red. Then, the threshold of the image was adjusted such that only dyed portion of the skin was captured, and all samples were processed using the same threshold value. The LTR area was quantified using image J software (National Institute of Health, USA) and the “Analyze Particles” option. The reported values were converted from units of pixels to mm using standards of known dimensions^7^.

### Calcein Permeability through the Skin

After treatment of the skin with LFU, the receiver chamber of each sample was filled with PBS (12 mL), and the donor chamber was filled with 2.5 mL of 0.2% calcein (Sigma-Aldrich, USA) in PBS. The receiver chambers were magnetically stirred at 400 rpm. The receiver solution was sampled at 2-h intervals between 18 and 26 h^15^. For each sample, a 1-mL aliquot was withdrawn from the receiver chamber and immediately replaced with an equal volume of PBS. The concentration of calcein in each aliquot was determined using a UV-visible spectrophotometer UV 1800 (Shimadzu, Japan) to measure absorbance at λ = 494 nm. This value was converted to units of % using a standard curve generated from known concentrations of calcein^6^. The skin permeability (P) of calcein at the steady-state was calculated using the equation P = (V/A.C_o_)/ΔC/Δt), where V (12 mL) is the volume of the receptor solution, A (1.13 cm^2^) is the area of the skin available for diffusion, C_o_ is the initial concentration (2 mg/cm^3^), and ΔC/Δt is the rate of change in the calcein concentration in the receptor solution at the steady-state.

### *In vitro* DOX penetration studies

After pre-treatment of skin with LFU/hydrogel, poloxamer gel 20% containing 400 μg/mL DOX (Zodiac, Brazil) was placed into contact with the pre-treated skin. The receiver chamber was filled with isotonic buffer (25 mM HEPES, 133 mM NaCl), pH 7.4 and magnetically stirred at 300 rpm. Samples were sampled at 2-h intervals between 18 and 26 hours. The amount of DOX in receiver solution after 26 h was analyzed by HPLC. The area of the skin exposed to DOX formulation was carefully collected. Then, the SC was tape-stripped using 15 tapes (Durex 18 mm, Brazil). The tape strips were subsequently immersed in 5 mL of methanol to extract the drug, and this solution was analyzed by HPLC to determine the amount of DOX in the SC. The remaining skin (“viable epidermis”) was cut into small pieces, and 5 mL of methanol was added. The skin was homogenized using a tissue homogenizer (Ultra Turrax IKA T 25, Germany) for 1 min, and an aliquot of the filtered homogenate was analyzed by HPLC to determine DOX in the “viable epidermis”.

### Analytical chemistry

DOX was quantified according to ref. 44 using a high-performance liquid chromatography system (Model LC10-AD, Shimadzu, Japan) consisting of pumps (LC10-AT), an automatic injector (model 9SIL-10AD), an oven (model CTO-10SA), a LiChrospher 100 RP-18 column (5 μm × 125 mm × 4 mm) and a guard column (5 μm × 10 mm × 4 mm) coupled to a fluorescence detector (model RF-10AX) and a computer equipped with chromatographic analysis software (CLASS-VP). The mobile phase consisted of phosphate buffer (50 mM, pH 2), acetonitrile and isopropanol (65:25:2) (v/v). Elution was performed at a constant flow rate of 1.0 mL/min using an injection volume of 100 μL at 35 °C. The fluorescence intensities of the eluents were monitored at 480/560 nm (excitation/emission). DOX exhibited a retention time of 3.4 min and a linear calibration curve (123.1*x* + 2878.3; *r *=* *0.9998) over the concentration range of 250–8000 ng/mL. The intra- and inter-day precision and accuracy of the method presented a coefficient of variation (%CV) and a relative error (%*E*) of not greater than 4.2% and 4.4%, respectively^44^.

### Statistical analysis

The data were statistically analyzed using one-way ANOVA followed by post hoc analysis using Tukey’s test with p < 0.05 as the minimum level of significance (GraphPad Prism software, version 5.01, USA).

## Additional Information

**How to cite this article:** Pereira, T. A. *et al*. Hydrogel increases localized transport regions and skin permeability during low frequency ultrasound treatment. *Sci. Rep.* **7**, 44236; doi: 10.1038/srep44236 (2017).

**Publisher's note:** Springer Nature remains neutral with regard to jurisdictional claims in published maps and institutional affiliations.

## Figures and Tables

**Table 1:** Zeta potential of the formulations used as the coupling medium in the LFU treatment.

Coupling medium	Zeta potential (mV)
SLS solution	−13.8 ± 3.2
Poloxamer hydrogel	+5.7 ± 2.1
HEC hydrogel	+1.1 ± 0.1
Chitosan hydrogel	+73.9 ± 2.2
Carbopol hydrogel	−33.1 ± 1.7

**Table 2:** Viscosity and interfacial tension values of the coupling medium before and immediately after 1 min of LFU treatment.

Coupling medium	Viscosity (Pa.s)		Interfacial tension (mN.m^−1^)	
	Before LFU	After LFU	Before LFU	After LFU
SLS	9 × 10^−4^ ± 2 × 10^−5^	5 × 10^−4^ ± 3 × 10^−5^*	35.9 ± 0.1	34.6 ± 0.1*
Poloxamer	2.6 ± 0.4	12.6 ± 1.5*	41.6 ± 0.4^1^	—^#^
HEC	2.3 ± 0.1	0.4 ± 0.1*	60.6 ± 0.9^1,2^	55.4 ± 2.0*
Chitosan	3.4 ± 0.1	1.1 ± 0.3*	63.1 ± 2.9^1,2^	48.7 ± 0.8*
Carbopol	3.1 ± 0.0	0.3 ± 0.0*	60.8 ± 0.2^1,2^	49.5 ± 0.7*

Values reported are Mean ± SD of 3 determinations for 3 different formulations. *Student’s paired t-test with p < 0.05 for viscosity or interfacial tension before the LFU treatment. ^1^p < 0.05 for SLS solution, ^2^p < 0.05 for poloxamer hydrogel. The results were analyzed using ANOVA followed by Tukey post hoc test with p < 0.05 as level of significance. ^#^It was not possible to measure the viscosity of the Poloxamer gel, a thermoreversible hydrogel, due to its high viscosity after 1 minute of FLU treatment.
